# Efficacy and safety of pegfilgrastim biosimilar MD‐110 in patients with breast cancer receiving chemotherapy: Single‐arm phase III

**DOI:** 10.1002/cam4.6519

**Published:** 2023-10-12

**Authors:** Toshimi Takano, Mitsuya Ito, Takayuki Kadoya, Tomofumi Osako, Tomoyuki Aruga, Norikazu Masuda, Toshiko Miyaki, Naoki Niikura, Daisuke Shimizu, Yuichi Yokoyama, Manabu Watanabe, Masato Tomomitsu, Kenjiro Aogi

**Affiliations:** ^1^ Breast Medical Oncology The Cancer Institute Hospital of Japanese Foundation for Cancer Research Tokyo Japan; ^2^ Department of Breast Surgery Hiroshima City Hiroshima Citizens Hospital Hiroshima Japan; ^3^ Department of Breast Surgery Hiroshima University Hospital Hiroshima Japan; ^4^ Breast Center Kumamoto Shinto General Hospital Kumamoto Japan; ^5^ Department of Breast Surgery Tokyo Metropolitan Center and Infectious Diseases Center Komagome Hospital Tokyo Japan; ^6^ Department of Surgery, Breast Oncology National Hospital Organization Osaka National Hospital Osaka Japan; ^7^ Department of Breast Surgery Chiba Cancer Center Chiba Japan; ^8^ Department of Breast Oncology Tokai University Hospital Isehara Japan; ^9^ Breast Center Yokohama City Minato Red Cross Hospital Yokohama Japan; ^10^ Mochida Pharmaceutical Co., Ltd. Tokyo Japan; ^11^ Department of Breast Surgery National Hospital Organization Shikoku Cancer Center Matsuyama Japan; ^12^ Present address: Department of Breast and Endocrine Surgery Nagoya University Graduate School of Medicine Nagoya Japan

**Keywords:** biosimilar, breast cancer, chemotherapy, febrile neutropenia, pegfilgrastim

## Abstract

**Introduction:**

Pegfilgrastim is indicated to decrease the incidence of chemotherapy‐induced febrile neutropenia. It is the first granulocyte‐colony stimulating factor approved for prophylactic use regardless of carcinoma type and is marketed in Japan as G‐LASTA (Kyowa Kirin Co., Ltd., Tokyo, Japan). MD‐110 is a biosimilar of pegfilgrastim. This phase III, multicenter, open‐label, single‐arm study investigated the efficacy and safety of MD‐110 in early‐stage breast cancer patients receiving neoadjuvant or adjuvant myelosuppressive chemotherapy.

**Methods:**

A total of 101 patients received the study drug. Each patient received docetaxel 75 mg/m^2^ and cyclophosphamide 600 mg/m^2^ (TC) for four cycles on day 1 of each cycle. MD‐110 (3.6 mg) was administered subcutaneously on day 2 of each cycle. The primary efficacy endpoint was the duration of severe neutropenia during cycle 1 (days with absolute neutrophil count < 500/mm^3^). The safety endpoints were adverse events and the presence of antidrug antibodies.

**Results:**

The mean (SD) duration of severe neutropenia for MD‐110 was 0.2 (0.4) days. The upper limit of the two‐sided 95% confidence interval for the mean duration of severe neutropenia was 0.2 days, below the predefined threshold of 3.0 days. The incidence of febrile neutropenia, the secondary efficacy endpoint, was 6.9% (7/101). Adverse events, occurring in more than 50% of patients, were alopecia, constipation, and malaise, which are common side effects of TC chemotherapy. Antidrug antibodies were negative in all patients.

**Conclusion:**

MD‐110 was effective against chemotherapy‐induced neutropenia. No additional safety concern, compared with the originator, was observed in patients with breast cancer receiving TC chemotherapy.(JapicCTI‐205230).

## Graphical abstract

Phase III study of MD‐110, a biosimilar of pegfilgrastim, was conducted to investigate the efficacy and safety in early‐stage breast cancer patients receiving neoadjuvant or adjuvant myelosuppressive chemotherapy. MD‐110 was effective against chemotherapy‐induced neutropenia. No additional safety concern, compared with the originator, was observed in patients with breast cancer receiving TC chemotherapy.

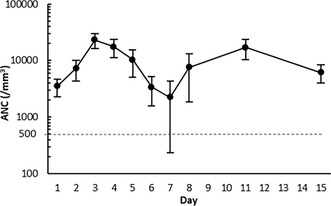

## 1 INTRODUCTION

Febrile neutropenia (FN) is the most common life‐threatening complication of myelosuppressive cancer chemotherapy. It is a major cause of morbidity, health care resource use and compromised treatment efficacy resulting from delays and dose reductions of cancer chemotherapy.^1^ FN is defined by the degree of fever and the peripheral blood absolute neutrophil count (ANC). According to the Japanese Society of Medical Oncology guidelines, FN is defined as “axillary body temperature ≥37.5°C or oral temperature ≥38°C and neutrophil count <500/mm^3^ or current neutrophil count <1000/mm^3^ and expected to fall below 500/mm^3^ during the next 48 h”.^2^

Pegfilgrastim is a PEGylated form of filgrastim and is indicated to decrease the incidence of chemotherapy‐induced FN. While filgrastim is administered daily, pegfilgrastim prevents chemotherapy‐induced FN by once‐per‐chemotherapy‐cycle dosing. It is the first granulocyte colony‐stimulating factor (G‐CSF) approved for prophylactic use regardless of carcinoma type. The Japan Society of Clinical Oncology strongly recommends its use over daily filgrastim injection.^3^ Pegfilgrastim is marketed as G‐LASTA (Kyowa Kirin Co., Ltd., Tokyo) in Japan and Neulasta (Amgen Inc., Thousand Oaks, CA) in other countries. The approved dose for chemotherapy‐induced FN is 3.6 mg in Japan and 6.0 mg in other countries. This difference is due to the similar efficacy results of 3.6 mg–6.0 mg observed in Japanese cancer patients.^4^, ^5^

Biological medicines are more expensive than small‐molecule medicines. Since the use of this therapy imposes a heavy financial burden on patients, less expensive biosimilars (BSs) have attracted attention as alternatives. Currently, there is no pegfilgrastim BS approved in Japan. MD‐110 was developed as the first BS to the originator, pegfilgrastim. Its active ingredient has an amino acid sequence bound to a methoxy polyethylene glycol (molecular weight: ca. 20,000) at N‐terminal methionine via linker, which is identical to that of the originator. Also, it has a high level of similarity to the originator in quality characteristics, pharmacological effects, and toxicities. Pharmacokinetic and pharmacodynamic bioequivalence between MD‐110 and the originator has been confirmed in healthy volunteers.

This phase III, multicenter, open‐label, single‐arm study investigated the efficacy and safety of MD‐110 in patients with breast cancer receiving cancer chemotherapy.

## 2 MATERIAL AND METHODS

### 2.1 Study design and treatment

**FIGURE 1:**
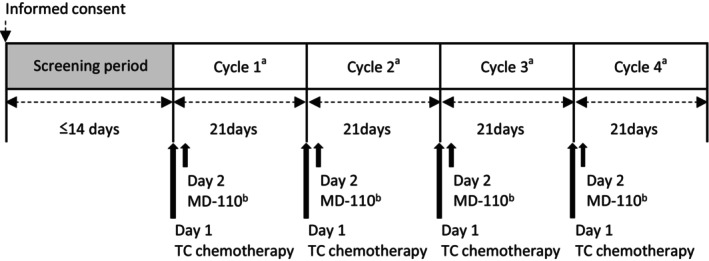
Study design. TC, docetaxel and cyclophosphamide. ^a^Study visits: Cycle 1 (Days 1–8, 11, and 15), Cycles 2 to 4 (Days 1 and 2), and End of study visit; ^b^MD‐110 was subcutaneously administered.

This was a multicenter, open‐label, single‐arm study designed to confirm the efficacy and safety of MD‐110. The study design is shown in Figure 1. Breast cancer patients scheduled to receive neoadjuvant or adjuvant cancer chemotherapy were eligible for the study. Eligible patients underwent four planned docetaxel and cyclophosphamide (TC) chemotherapy cycles every 21 days, the same chemotherapy regimen employed in the originator study. In each cycle, TC chemotherapy was administered on Day 1, and a single subcutaneous 3.6 mg dose of MD‐110 was administered on Day 2 (at least 24 h after the end of chemotherapy). In cycle 1, TC chemotherapy was administered at the standard dose (docetaxel 75 mg/m^2^ and cyclophosphamide 600 mg/m^2^). A 20% dose reduction of the TC chemotherapy was permitted for the purpose of mitigating the chemotherapy‐induced side effects. TC chemotherapy was selected because of its high risk of FN and wide use in Japanese patients with early‐stage breast cancer. Guidelines recommend G‐CSF as the primary prophylaxis of chemotherapy‐induced neutropenia when the overall risk of FN for the prescribed regimen is over 20%.^2^, ^6^, ^7^, ^8^

Medications that affect the efficacy and safety evaluation of MD‐110 were prohibited during the study. Antibiotics, antimicrobials, antipyretic analgesics, and traditional Chinese medicine were permitted for a fever over 37.5°C or pain. Corticosteroids were also permitted to prevent nausea and vomiting caused by TC chemotherapy.

### 2.2 Patients

Female patients with a clinical or pathologic diagnosis of stage I‐III primary invasive breast cancer were enrolled. Other key inclusion criteria were as follows: (i) ≥20 and < 70 years of age; (ii) scheduled to receive 4 cycles of neoadjuvant or adjuvant TC chemotherapy; (iii) ECOG performance status ≤2; (iv) ANC ≥1500/mm^3^.

### 2.3 Endpoints

The primary efficacy endpoint was the duration of severe (grade 4) neutropenia (DSN) during cycle 1.^9^, ^10^ DSN was defined as the number of consecutive days with an ANC < 500/mm^3^. The secondary efficacy endpoint was the incidence of FN across all cycles and at cycle 1.^9^, ^10^ FN was defined as a fever of 37.5°C or higher within 1 day before or after of an ANC measurement of < 500/mm^3^.

ANC measurement was scheduled for days 1–8, 11, and 15 of cycle 1 (10 time points in total). If ANC was <1000/mm^3^, additional ANC measurement was undertaken every day until recovery to ≥1000/mm^3^. In subsequent cycles, ANC was scheduled for days 1 and 2 (two time points in total). This is twice as many measurements as the originator study for cycle 1 but half as many for subsequent cycles. In the originator study, measurements were taken on days 1, 2, 8, 11, and 15 of cycle 1 and days 1, 2, 8, and 11 of subsequent cycles. Body temperature was recorded daily, as was done in the originator study. Patients with a fever of 37.5°C or higher were requested to visit the medical institution within 1 day for ANC measurement, as was done in the originator study.

The safety endpoints were adverse events (AEs) across all cycles and the presence of antidrug antibodies (ADAs). AEs were defined by the Japanese version of the Medical Dictionary for Regulatory Activities (MedDRA) (version 24.1) and graded using the Common Terminology Criteria for Adverse Events (CTCAE) (version 5.0).

### 2.4 Statistical analysis

Based on the results of several studies, the effect size and standard deviation (SD) of the DSN for the originator (pegfilgrastim 3.6 mg) were estimated to be 1.9 days and 1.4 days, respectively. The DSN threshold value was set at 3.0 days by adding 1.0 days, about one‐half of 1.9 days, to 2.0 days, which is the mean DSN for the originator. In terms of efficacy, 20 patients were needed to ensure that the probability of the upper limit of a two‐sided 95% confidence interval (CI) for the mean DSN being less than the threshold is greater than 90%. On the contrary, from the safety viewpoint, 100 patients were required to detect at least one adverse event whose incidence is 3% or higher with a 95% probability. The target sample size of 100 patients was determined by both safety and efficacy evaluations.

Efficacy analyses were performed on the full analysis set (FAS), consisting of patients who received at least one dose of MD‐110 with at least one postbaseline ANC. The primary efficacy endpoint, DSN, was analyzed by descriptive statistics and a two‐sided 95% CI of the mean. DSN for cycle 1 withdrawn patients with the last ANC of <500/mm^3^ or ≥ 500/mm^3^ with no nadir were imputed with the maximum DSN value calculated from patients completing all scheduled ANC measurements in cycle 1. The secondary efficacy endpoint, FN, was analyzed by calculating the incidence rate.

Safety analyses were performed on the safety analysis set (SS), consisting of patients who received at least one dose of MD‐110 with postbaseline safety data. AEs were analyzed by calculating the incidence rate. All statistical analyses were performed using SAS Release 9.4 (SAS Institute Inc., Cary, NC, USA).

## 3 RESULTS

### 3.1 Patients

**FIGURE 2:**
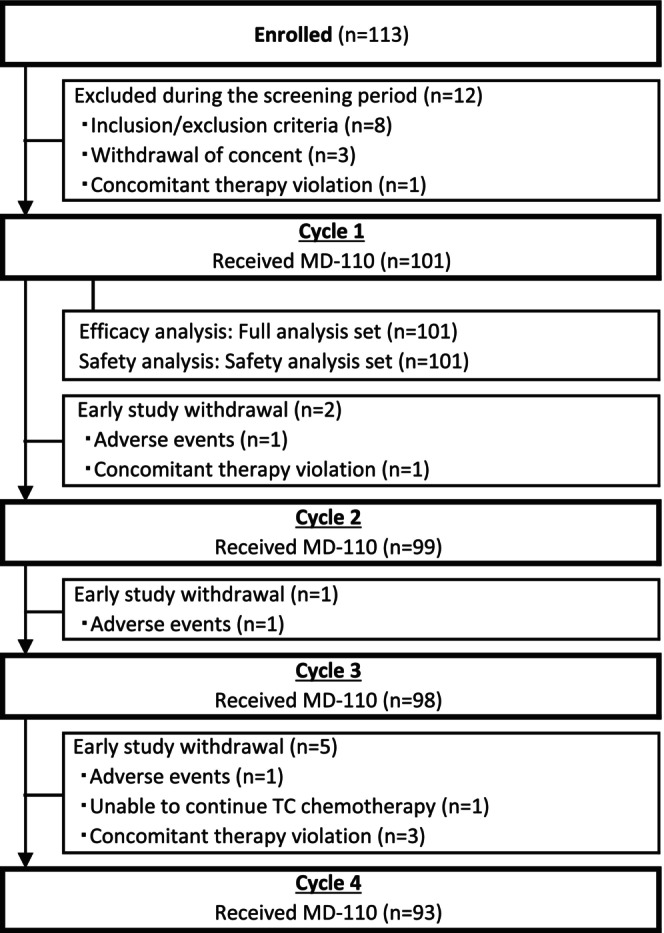
Patient disposition.

This study was conducted at 24 sites in Japan. A total of 113 patients were enrolled in the study, of whom 101 received the study drug and were included in the FAS and SS. The patient disposition is shown in Figure 2. Regarding cancer chemotherapy, of the 93 patients who completed all 4 cycles, 92.5% (86/93) of patients received TC chemotherapy at the standard dose. The percentage of patients whose TC chemotherapy dose was reduced by 20% in cycles 2, 3, and 4 were 5.1% (5/99), 6.1% (6/98), and 7.5% (7/93), respectively. Out of the seven patients who had their TC chemotherapy dose reduced in total, three were attributed to hematologic toxicity events, including two cases with low ANC (below 1500/mm^3^) and one with a grade 3 decreased lymphocyte count. Patients with dose reduction were maintained on the reduced dose for the remaining cycles.

The baseline characteristics are shown in Table 1. The patients were all Asian females.

**TABLE 1:** Baseline characteristics (full analysis set).

Characteristics		MD‐110 (*n* = 101)
Age, years	Mean ± SD	51.9 ± 10.5
	Median (min‐max)	49.0 (29–69)
Age group, *n* (%)	<65	85 (84.2)
	≥65	16 (15.8)
BMI, kg/m^2^	Mean ± SD	22.32 ± 3.55
ECOG performance status, *n* (%)	0	101 (100.0)
Chemotherapy, *n* (%)	Neoadjuvant	1 (1.0)
	Adjuvant	100 (99.0)
TNM stage, *n* (%)	I	30 (29.7)
	IIA	41 (40.6)
	IIB	20 (19.8)
	IIIA	9 (8.9)
	IIIB	0
	IIIC	1 (1.0)
Previous radiotherapy, *n* (%)	No	96 (95.0)
	Yes	5 (5.0)

Abbreviations: BMI, body mass index; SD, standard deviation.

### 3.2 Efficacy

**FIGURE 3:**
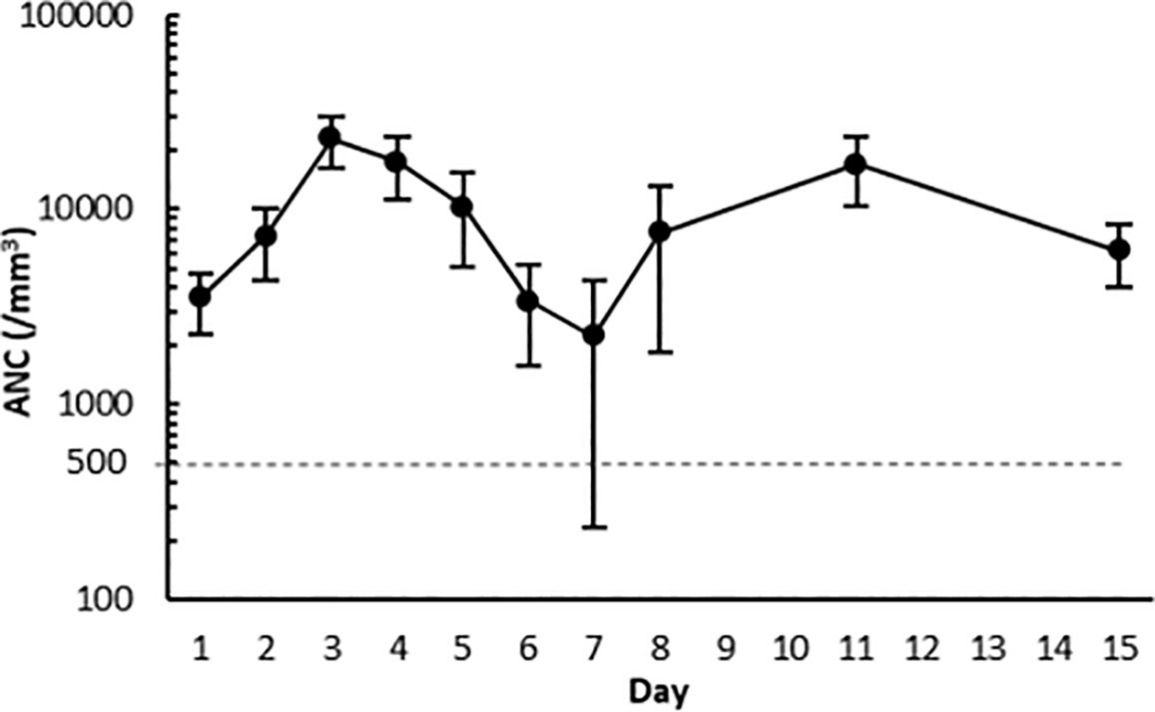
Changes in absolute neutrophil count in the cycle 1 (full analysis set). Data points and error bars indicate the mean and SD. ANC, absolute neutrophil count; SD, standard deviation.

The summary of efficacy endpoints is shown in Table 2. The mean (SD) DSN for cycle 1 was 0.2 (0.4) days (two‐sided 95% CI: 0.1–0.2 days). The upper limit of the two‐sided 95% CI for the mean DSN was 0.2 days, which was below the threshold of 3.0 days predefined in the study protocol. Mean ANC during cycle 1 was highest on Day 3 and lowest on Day 7 (Figure 3). One patient withdrew from cycle 1 with a final measurement of ANC ≥ 500/mm^3^, and the nadir was not determined. Based on the protocol, the DSN of this patient was imputed with “2 days”, which was the maximum DSN value calculated from patients completing all scheduled ANC measurements in cycle 1.

**TABLE 2:** Summary of efficacy endpoints (full analysis set).

Efficacy endpoints		MD‐110 (*n* = 101)
DSN Cycle 1	Mean (SD), days	0.2 (0.4)
	Mean, two‐sided 95% CI (lower limit–upper limit), days	0.1–0.2
	Median (min‐ max), days	0.0 (0–2)
	0 day, *n* (%)	87 (86.1)
	1 day, *n* (%)	12 (11.9)
	2 day, *n* (%)	2 (2.0)
FN All cycles	*n* (%)	7 (6.9)

Abbreviations: CI, confidence interval; DSN, duration of severe neutropenia; FN, febrile neutropenia; SD, standard deviation.

The details of patients with FN are shown in Table 3. FN occurred in seven patients. Of the seven patients, six occurred during cycle 1 and one during cycle 2. All six patients with FN during cycle 1 recorded ANC <500/mm^3^ and fever ≥37.5°C on Day 7, and ANC recovered to ≥500/mm^3^ on Day 8. Of the seven patients who developed FN, one patient had a fever of 38.7°C during cycle 2, equivalent to grade 1 in CTCAE v5.0 (38.0°C or higher).

**TABLE 3:** Details of patients with febrile neutropenia (full analysis set).

No.	Age (year)	Time point		ANC (/mm^3^)	Body temperature(°C)
1	69	Cycle 1	Day 2^a^	9700	36.5
			Day 6	700	36.8
			Day 7^b^	100	37.8
			Day 8	2900	36.8
2	63	Cycle 1	Day 2^a^	4985	36.4
			Day 6	1600	37.5
			Day 7^b^	469	37.7
			Day 8	4247	36.8
3	38	Cycle 1	Day 2^a^	4370	36.7
		Cycle 2	Day 6	–^c^	37.1
			Day 7^b^	260	38.7
			Day 8	–^c^	36.7
		Cycle 3	Day 1	2660	36.1
4	69	Cycle 1	Day 2^a^	6742	36.7
			Day 6	2206	37.3
			Day 7^b^	369	37.5
			Day 8	6304	36.8
5	44	Cycle 1	Day 2^a^	9340	37.3
			Day 6	780	37.4
			Day 7^b^	100	37.9
			Day 8	1490	37.5
6	50	Cycle 1	Day 2^a^	8000	36.2
			Day 6	600	37.0
			Day 7^b^	400	37.5
			Day 8	4700	36.3
7	61	Cycle 1	Day 2^a^	9800	36.1
			Day 6	500	36.8
			Day 7^b^	200	37.8
			Day 8	2900	37.6

Abbreviations: ANC, absolute neutrophil count.

^a^ Day 2 of Cycle 1 represents the baseline of ANC and body temperature.

^b^ The day FN occurred.

^c^ ANC was not scheduled.

### 3.3 Safety

The summary of AEs is shown in Table 4. There were no AEs leading to death. Serious AEs (SAEs) occurred in six patients, including urticaria, decreased performance status, diverticulitis, pneumonia, rash, and malaise (one SAE per patient). Among these SAEs, urticaria was the only SAE related to the study drug. However, it did not lead to study withdrawal and recovered with antihistamine treatment and was considered to be caused by the combination of TC chemotherapy and the study drug. Three patients withdrew from the study due to pneumonia, decreased appetite, and urticaria (one AE per patient). Study drug‐related AEs of grade 3 or higher were urticaria in two patients and increased alanine aminotransferase in one patient. One case of urticaria was a study drug‐related SAE. The remaining two cases were nonserious, did not lead to study withdrawal, and recovered with drug treatment.

**TABLE 4:** Summary of adverse events across all cycles (safety analysis set).

Category	MD‐110 (*n* = 101)	
	AEs *n* (%)	AEs related to study drug *n* (%)
All AEs	101 (100.0)	55 (54.5)
Grade ≥ 3 AEs	48 (47.5)	3 (3.0)
AEs leading to death	0	0
Serious AEs besides death	6 (5.9)	1 (1.0)
AEs leading to study withdrawal	3 (3.0)	0

Abbreviations: AE, adverse event.

Adverse events, occurring in more than 50% of patients, were common side effects of TC chemotherapy, and therefore the relationships with the study drug were mostly ruled out (Table S1). Study drug‐related AEs occurring at least 10% of patients were back pain (24.8%, 25/101), arthralgia (17.8%, 18/101), and pyrexia (10.9%, 11/101) (Table S2).

The incidence of AEs in cycles 1–4 was 100.0% (101/101), 92.9% (92/99), 87.8% (86/98), and 86.0% (80/93), respectively. The incidence of study drug‐related AEs in cycles 1–4 was 45.5% (46/101), 27.3% (27/99), 22.4% (22/98), and 16.1% (15/93), respectively. Pyrexia, a study drug‐related AE, gradually increased in frequency during the course of chemotherapy. The incidence of pyrexia in cycles 1–4 was 3.0% (3/101), 6.1% (6/99), 6.1% (6/98), and 6.5% (6/93), respectively. All cases were nonserious, grade 1 or grade 2 in severity, did not lead to study withdrawal, and recovered. Pyrexia did not tend to worsen in severity over chemotherapy cycles.

We calculated the incidence of adverse events of special interest (AESIs) based on the risk management plan of the originator (Table 5). Most AESIs did not occur in this study. Study drug‐related AESIs that occurred in this study were bone pain/back pain, interstitial lung disease, and blast cell count increase. Bone pain/back pain were all nonserious, grade 1 or grade 2 in severity, and did not lead to study withdrawal. Some patients with bone pain/back pain required antipyretic analgesic treatment. Interstitial lung disease occurred in one patient (1.0%). It was nonserious, grade 2 in severity, did not lead to study withdrawal, and recovered with oral corticosteroid treatment. Although it was considered strongly related to docetaxel, the causal relationship with the study drug was not ruled out. Increase in blast cell count, described as “myeloblast present” in Table S2, was observed in one patient (1.0%) with a count of 1.0%. It was nonserious, grade 1 in severity, and the patient did not require study withdrawal or any treatment. Although it was considered to be caused by TC chemotherapy, a causal relationship with the study drug was not ruled out.

**TABLE 5:** Adverse events of special interest across all cycles (safety analysis set).

Category	MD‐110 (*n* = 101)	
	AESIs *n* (%)	AESIs related to study drug *n* (%)
Bone pain/back pain^a^	85 (84.2)	43 (42.6)
Interstitial lung disease^b^	5 (5.0)	1 (1.0)
Blast cell count increased	1 (1.0)	1 (1.0)
Shock/anaphylactic shock	2 (2.0)	0
Serious platelet count decreased (CTCAE ≥3^c^)	1 (1.0)	0
Splenomegaly/splenic rupture	0	0
Acute respiratory distress syndrome	0	0
Capillary leak syndrome	0	0
Sweet syndrome	0	0
Cutaneous vasculitis	0	0
Large vessel vasculitis (aorta, common carotid artery, and subclavian artery)	0	0
Second primary malignancy	0	0

Abbreviations: AESIs, adverse events of special interest; CTCAE, common terminology criteria for adverse events.

^a^ AESIs classified as “Musculoskeletal and connective tissue disorders” by MedDRA System Organ Class.

^b^ AESIs classified as “interstitial lung disease”, “dyspnea”, “dyspnea exertional”, “lung disorder”, and “pneumonitis” by MedDRA Preferred Term and “dry cough” by MedDRA Lowest Level Term;

^c^ CTCAE grade 3 is defined as platelet count of <50000/mm^3^.

Regarding immunogenicity assessment, ADAs were negative in all patients.

## 4 DISCUSSION

While our single‐arm study did not include a direct comparison of MD‐110 and the originator, the results of the primary endpoint suggest that MD‐110 is comparable in efficacy to the originator in terms of DSN. The median DSN was 0 days (86.1% of patients had a DSN of 0 days), meaning that most patients' ANC did not drop below 500/mm^3^. In all cases where ANC fell to <500 /mm^3^, the value returned to ≥500/mm^3^ within 2–3 days. The ANC on days 1, 2, 8, 11, and 15 were similar to those observed in the originator.^11^ These results show that MD‐110 is effective against chemotherapy‐induced neutropenia.

The FN incidence rate in this study was 6.9% (7/101) for all cycles, slightly higher than the FN incidence rate reported in the originator study, which was 1.2% (2/173). Notably, a significant difference in the FN incidence rate was observed in the first cycle, with our study reporting a rate of 6.0% (6/101) compared to 0.5% (1/173) in the originator study. Since our study excluded patients at risk for increased FN frequency, such as those with hematopoietic dysfunction, advanced cancer, or serious complications (renal or hepatic dysfunction), we attribute this difference to the higher frequency of ANC measurements in our study. Our study required ANC measurements on days 1–8, 11, and 15 of cycle 1 (10 times in total), whereas the originator study only had five measurements on Days 1, 2, 8, 11, and 15. Upon closer examination of the six cases of FN in the first cycle of our study, we found that ANC levels below 500/μL were only observed on Day 7 (Table 3), which was not a specified measurement day in the originator study. If we had used the same ANC measurement frequency as the originator study, these six cases of FN in the first cycle might have gone undetected.

The incidence of FN in TC‐treated breast cancer patients with pegfilgrastim ranged from 0% to 10% and varied from study to study.^11^, ^12^, ^13^, ^14^, ^15^, ^16^, ^17^, ^18^ This difference may be influenced by differences in the definition of FN in various guidelines and the frequency of ANC measurements employed.^1^, ^2^, ^8^, ^19^, ^20^ Despite our study's high‐frequency ANC measurement, the FN incidence of 6.9% across all cycles falls within this range.

There was no additional safety concern observed compared with the originator. No unexpected SAEs related to the study drug occurred. Among study drug‐related AEs that occurred in at least 10% of patients (back pain, arthralgia, and pyrexia), the incidence of back pain (24.8%, 25/101) was slightly higher than that associated with the originator (17.3%, 30/173).^21^ However, all cases were nonserious and did not lead to study withdrawal; furthermore, all cases recovered. Interstitial lung disease was observed in one patient (1.0%) who recovered with oral corticosteroid treatment. Interstitial lung disease is reported as serious pneumonitis for the originator.^5^ A blast cell count increase was observed in one patient (1.0%) who recovered without treatment. The increase in blast cells with a count of 1.0% was considered to reflect the mobilization of hematopoietic stem cells into the peripheral blood. Blast cell count increase is reported as a drug‐related SAE in patients with breast cancer receiving the originator.^22^

ADAs were negative in this study, whereas the presence of ADAs has been reported for the originator.^21^

It is important to note several limitations of this study. Firstly, the study design was not a randomized controlled trial. Secondly, excluding patients aged 70 years or older may limit the generalizability of the findings to older populations. Thirdly, the small sample size may have limited the ability to detect rare adverse events previously reported with pegfilgrastim. Additionally, given the focus on a single cancer type and chemotherapy regimen, the efficacy and safety of MD‐110 in other cancers and treatment settings remain unknown. In order to fully assess the potential of MD‐110 in a broader range of patients, further research should explore its use in different cancers and treatment regimens, as well as with more diverse patient populations.

In conclusion, this study demonstrated that MD‐110 is effective against chemotherapy‐induced neutropenia with no additional safety concern compared with the originator in patients with breast cancer receiving TC chemotherapy. MD‐110 is expected to be the first pegfilgrastim BS approved in Japan for prophylactic use regardless of carcinoma type. MD‐110 may help fill an unmet need by providing greater affordability compared to existing treatment.

## AUTHOR CONTRIBUTIONS

**Toshimi Takano:** Conceptualization (equal); investigation (equal); writing – original draft (lead). **Mitsuya Ito:** Investigation (equal); writing – review and editing (equal). **Takayuki Kadoya:** Investigation (equal); writing – review and editing (equal). **Tomofumi Osako:** Investigation (equal); writing – review and editing (equal). **Tomoyuki Aruga:** Investigation (equal); writing – review and editing (equal). **Norikazu Maduda:** Investigation (equal); writing – review and editing (equal). **Toshiko Miyaki:** Investigation (equal); writing – review and editing (equal). **Naoki Niikura:** Investigation (equal); writing – review and editing (equal). **Daisuke Shimizu:** Investigation (equal); writing – review and editing (equal). **Yuichi Yokoyama:** Conceptualization (equal); data curation (equal); formal analysis (equal); methodology (equal); software (equal); visualization (equal); writing – original draft (equal). **Manabu Watanabe:** Conceptualization (equal); data curation (equal); methodology (equal); project administration (equal); supervision (equal); writing – original draft (equal). **Masato Tomomitsu:** Conceptualization (equal); methodology (equal); resources (equal); supervision (equal); writing – review and editing (equal). **Kenjiro Aogi:** Investigation (equal); writing – review and editing (equal).

## FUNDING INFORMATION

This study was funded by Mochida Pharmaceutical Co., Ltd. The study drug was provided by Mochida Pharmaceutical Co., Ltd.

## CONFLICT OF INTEREST STATEMENT

T.T. received honoraria from Celltrion, Chugai, Daiichi Sankyo, Eisai, and Eli Lilly. M.I., T.K., T.O., T.M., D.S., and K.A. have no conflict of interest. T.A. received honoraria from AstraZeneca, Chugai, Eisai, Eli Lilly, Kyowa Kirin, and Pfizer. N.M received honoraria from AstraZeneca, Chugai, Eisai, Eli Lilly, and Pfizer; and research funds from AstraZeneca, Chugai, Daiichi Sankyo, Eisai, Eli Lilly, Kyowa Kirin, Mochida, MSD, Nihon Kayaku, Novartis, Pfizer, and Sanofi. N.N. received honoraria from AstraZeneca, Chugai, Daiichi Sankyo, Eisai, Eli Lilly Japan, Nippon Kayaku, and Pfizer; and research funds from Chugai, Daiichi Sankyo, Eli Lilly Japan, Mochida, Nippon Kayaku, and Pfizer; and research grants from Chugai and Eisai. Y.Y., M.W., and M.T. are Mochida Pharmaceutical Co., Ltd employees.

## ETHICS STATEMENT

The study protocol was approved by the Ethics committee or the Institutional Review Board at each medical institution. All patients provided signed informed consent before the start of any study‐related procedures. The study was conducted in accordance with the International Council for Harmonization Good Clinical Practice Guideline and conformed to the provisions of the Declaration of Helsinki. The study was registered at Japanese Pharmaceutical Information Center Clinical Trials Information, number JapicCTI‐205230. Animal studies: N/A.

## Supporting information


Tables S1–S2

## Data Availability

The author elects to not share data.
