# Supplementary material for: Efficacy and safety of pegfilgrastim biosimilar MD‐110 in patients with breast cancer receiving chemotherapy: Single‐arm phase III
Source: Cancer Med. 2023 Oct 12;12(20):20242–50. doi: 10.1002/cam4.6519 (PMC10652341; doi:10.1002/cam4.6519)
Supplement: Supplementary file 1 — Tables S1–S2 [file CAM4-12-20242-s001.docx]

**TABLE S1** Adverse events occurring in ≥ 10% of patients across all cycles (safety analysis set)

| AEs (≥ 10%) | MD-110 (n=101) n (%) | | | | |
| --- | --- | --- | --- | --- | --- |
|  | All grades | Grade 1 | Grade 2 | Grade 3 | Grade 4 |
| Alopecia | 89(88.1) | 19(18.8) | 70(69.3) | 0 | 0 |
| Constipation | 58(57.4) | 45(44.6) | 13(12.9) | 0 | 0 |
| Malaise | 57(56.4) | 51(50.5) | 3(3.0) | 3(3.0) | 0 |
| Nausea | 47(46.5) | 30(29.7) | 17(16.8) | 0 | 0 |
| Arthralgia | 45(44.6) | 27(26.7) | 18(17.8) | 0 | 0 |
| Taste disorder | 44(43.6) | 44(43.6) | 0 | 0 | 0 |
| Back pain | 39(38.6) | 28(27.7) | 11(10.9) | 0 | 0 |
| Pyrexia | 39(38.6) | 34(33.7) | 5(5.0) | 0 | 0 |
| Myalgia | 36(35.6) | 24(23.8) | 12(11.9) | 0 | 0 |
| Diarrhea | 34(33.7) | 27(26.7) | 7(6.9) | 0 | 0 |
| Edema peripheral | 33(32.7) | 22(21.8) | 11(10.9) | 0 | 0 |
| Headache | 30(29.7) | 19(18.8) | 11(10.9) | 0 | 0 |
| Neutrophil count decreased | 30(29.7) | 0 | 9(8.9) | 11(10.9) | 10(9.9) |
| Stomatitis | 23(22.8) | 16(15.8) | 7(6.9) | 0 | 0 |
| Urticaria | 23(22.8) | 8(7.9) | 11(10.9) | 4(4.0) | 0 |
| Rash | 22(21.8) | 10(9.9) | 11(10.9) | 1(1.0) | 0 |
| Pruritus | 20(19.8) | 14(13.9) | 6(5.9) | 0 | 0 |
| White blood cell count decreased | 19(18.8) | 2(2.0) | 6(5.9) | 10(9.9) | 1(1.0) |
| Decreased appetite | 19(18.8) | 15(14.9) | 3(3.0) | 1(1.0) | 0 |
| Anemia | 18(17.8) | 9(8.9) | 9(8.9) | 0 | 0 |
| Lymphocyte count decreased | 18(17.8) | 0 | 4(4.0) | 12(11.9) | 2(2.0) |
| Peripheral sensory neuropathy | 18(17.8) | 14(13.9) | 4(4.0) | 0 | 0 |
| Eczema | 17(16.8) | 5(5.0) | 12(11.9) | 0 | 0 |
| Edema | 16(15.8) | 13(12.9) | 3(3.0) | 0 | 0 |
| Insomnia | 13(12.9) | 10(9.9) | 3(3.0) | 0 | 0 |
| Neuropathy peripheral | 13(12.9) | 12(11.9) | 1(1.0) | 0 | 0 |
| Platelet count decreased | 13(12.9) | 9(8.9) | 3(3.0) | 1(1.0) | 0 |
| Vomiting | 12(11.9) | 9(8.9) | 3(3.0) | 0 | 0 |
| Palmar-plantar erythrodysesthesia syndrome | 11(10.9) | 8(7.9) | 3(3.0) | 0 | 0 |

Abbreviations: AE, adverse event.

**TABLE S2** Study drug-related adverse events across all cycles (safety analysis set)

| Study drug-related AEs | MD-110 (n=101) n (%) | | | |
| --- | --- | --- | --- | --- |
|  | All grades | Grade 1 | Grade 2 | Grade 3 |
| Back pain | 25(24.8) | 19(18.8) | 6(5.9) | 0 |
| Arthralgia | 18(17.8) | 9(8.9) | 9(8.9) | 0 |
| Pyrexia | 11(10.9) | 9(8.9) | 2(2.0) | 0 |
| Headache | 9(8.9) | 8(7.9) | 1(1.0) | 0 |
| Urticaria | 7(6.9) | 2(2.0) | 3(3.0) | 2(2.0) |
| Blood lactate dehydrogenase increased | 5(5.0) | 5(5.0) | 0 | 0 |
| Myalgia | 5(5.0) | 3(3.0) | 2(2.0) | 0 |
| Bone pain | 4(4.0) | 3(3.0) | 1(1.0) | 0 |
| C-reactive protein increased | 4(4.0) | 4(4.0) | 0 | 0 |
| Malaise | 4(4.0) | 4(4.0) | 0 | 0 |
| Rash | 3(3.0) | 2(2.0) | 1(1.0) | 0 |
| Alanine aminotransferase increased | 2(2.0) | 1(1.0) | 0 | 1(1.0) |
| Blood alkaline phosphatase increased | 2(2.0) | 2(2.0) | 0 | 0 |
| Metamyelocyte count | 2(2.0) | 2(2.0) | 0 | 0 |
| Myelocyte count | 2(2.0) | 2(2.0) | 0 | 0 |
| Musculoskeletal discomfort | 2(2.0) | 1(1.0) | 1(1.0) | 0 |
| Pain | 2(2.0) | 0 | 2(2.0) | 0 |
| Pruritus | 2(2.0) | 1(1.0) | 1(1.0) | 0 |
| Aspartate aminotransferase increased | 1(1.0) | 0 | 1(1.0) | 0 |
| Eczema | 1(1.0) | 0 | 1(1.0) | 0 |
| Gamma-glutamyltransferase increased | 1(1.0) | 1(1.0) | 0 | 0 |
| Hepatic enzyme increased | 1(1.0) | 1(1.0) | 0 | 0 |
| Hepatic function abnormal | 1(1.0) | 0 | 1(1.0) | 0 |
| Interstitial lung disease | 1(1.0) | 0 | 1(1.0) | 0 |
| Myeloblast present | 1(1.0) | 1(1.0) | 0 | 0 |
| Neck pain | 1(1.0) | 0 | 1(1.0) | 0 |
| Oropharyngeal pain | 1(1.0) | 1(1.0) | 0 | 0 |
| Platelet count decreased | 1(1.0) | 0 | 1(1.0) | 0 |
| Platelet count increased | 1(1.0) | 1(1.0) | 0 | 0 |
| Toothache | 1(1.0) | 1(1.0) | 0 | 0 |

Abbreviations: AE, adverse event.
